# Decoding Musical Training from Dynamic Processing of Musical Features in the Brain

**DOI:** 10.1038/s41598-018-19177-5

**Published:** 2018-01-15

**Authors:** Pasi Saari, Iballa Burunat, Elvira Brattico, Petri Toiviainen

**Affiliations:** 1 Department of Music, Art, and Culture Studies, University of Jyväskylä, Jyväskylä, PL 35(M), FI-40014 Finland; 2 Center for Music in the Brain (MIB), Department of Clinical Medicine, Aarhus University, Aarhus, DK-8000 Denmark

## Abstract

Pattern recognition on neural activations from naturalistic music listening has been successful at predicting neural responses of listeners from musical features, and vice versa. Inter-subject differences in the decoding accuracies have arisen partly from musical training that has widely recognized structural and functional effects on the brain. We propose and evaluate a decoding approach aimed at predicting the musicianship class of an individual listener from dynamic neural processing of musical features. Whole brain functional magnetic resonance imaging (fMRI) data was acquired from musicians and nonmusicians during listening of three musical pieces from different genres. Six musical features, representing low-level (timbre) and high-level (rhythm and tonality) aspects of music perception, were computed from the acoustic signals, and classification into musicians and nonmusicians was performed on the musical feature and parcellated fMRI time series. Cross-validated classification accuracy reached 77% with nine regions, comprising frontal and temporal cortical regions, caudate nucleus, and cingulate gyrus. The processing of high-level musical features at right superior temporal gyrus was most influenced by listeners’ musical training. The study demonstrates the feasibility to decode musicianship from how individual brains listen to music, attaining accuracy comparable to current results from automated clinical diagnosis of neurological and psychological disorders.

## Introduction

Neural processing of naturalistic musical signals involves dynamic integration of a variety of musical features^1^. Continuous listening of music is thus effective at recruiting several fronto-temporal, parietal and limbic areas across the brain, showing the involvement of both perceptual, action-simulation, emotional and attentional processes during feature integration. A large number of features describing musical audio signals, developed in the field of Music Information Retrieval (MIR)^2^, can be computed using many available tools, such as MIR Toolbox^3^. These features include low-level musical features, i.e., features associated with early stages of sound processing, such as those related to pitch, timbre, and amplitude, and high-level features, such as those related to tonality, pulse, or song structure. In MIR, pattern recognition approaches have been employed to model semantic concepts, such as emotion or genre using combinations of musical features as inputs^4^. Combining these approaches makes it possible to model time-series of neural activations with musical features, and vice-versa. These methods are referred to as encoding and decoding, respectively^5^. Using naturalistic music as stimuli, recent studies have shown the advantages of encoding and decoding for describing the links between dynamic changes in the musical features and time courses of neural activations recorded using electroencephalography (EEG)^6–8^ and functional Magnetic Resonance Imaging (fMRI)^1,9–11^. Using fMRI, significant correlations have been discovered across the brain between musical features and the voxel-wise blood-oxygen-level dependent (BOLD) time-series^1^. The found correlations include those between low-level timbral features and cognitive areas of the cerebellum and sensory and default mode network cerebrocortical areas, and between high-level pulse and tonality features and cognitive, motor and emotion-related circuits. By combining multiple musical features for voxel-wise fMRI encoding by means of linear regression, significant prediction accuracy has been obtained for auditory, limbic, and motor regions, notably for medial orbitofrontal region, anterior cingulate cortex, and right superior temporal gyrus^9^. When it comes to the decoding of musical features from BOLD time-series, significant accuracy has been obtained for timbral and rhythmic features for the majority of participants^10^. However, the accuracy levels for the high-level key clarity feature varied between participants, which suggests high inter-participant variability in the neural processing.

Although participant-specific differences in the neural processing of music are a result of a multitude of demographical and background variables, musical expertise resulting from music instrument training has been identified as a major contributor. Only 15 months of musical training in early childhood leads to structural changes in the brain and may lead to improvements in musically relevant motor and auditory skills^12^. Motor demands of music instrument playing have been linked to functional symmetry in the brain regions involved in somatosensory and motor control in the parietal and frontal lobes^13^. This relates to neuroplasticity that enables the optimization of behavior to environmental demands^14^. Furthermore, an enhancing cross-modal transfer effect of musical training has been found on tasks requiring nonmusical auditory processing, such as speech and language learning and the evaluation of vocal expressions of emotion^15^.

Past studies on the effect of musical training on the brain have utilized statistical analyses of functional or structural group differences between musicians and nonmusicians^16–19^. However, the used approaches lack the capability of integrating dynamic relationships between multiple brain areas and musical features. Moreover, the used statistical techniques, usually yielding significance estimates as p-values, are not developed to inform about their generalizability to participant populations not involved in the study. In this study, we devised a multivariate pattern classification framework, evaluated by cross-validation with held-out participants, that overcomes these weaknesses. Instead of p-values of group differences, the performance is evaluated by means of classification accuracy, defined as the number of correctly classified participants divided by the number of evaluated participants. By shifting from using p-values to using classification accuracy, we change the question “how significant are the differences between musicians and nonmusicians” to the question “how likely would a person not used in the model training be identified correctly as a musician or nonmusician”. In general, it is more challenging to show a significant effect by means of classification accuracy than by statistical analysis of group differences^20^. Outside the musical domain, single subject prediction has received considerable attention in the automated clinical diagnosis of neurological and psychiatric disorders^20^. The approaches, mainly based on whole brain structural MRI or fMRI in the resting state or task dependent conditions, have reached high correct classification rates in separating patients from healthy controls^21–24^. According to a survey based on 200 studies focused on MRI-based single subject prediction, median classification accuracies obtained for different brain disorders have ranged from 75% (attention-deficit hyperactivity disorder, stable versus progressive mild cognitive impairment) to over 85% (Altzheimer’s disease, autism spectrum disease)^20^.

The present study is the first study to propose and evaluate an approach for decoding musical training in music neuroscience. The main novelty of the proposed decoding approach lies in combining computational acoustic feature extraction with neuroimaging data and using the temporal evolution of brain responses to music obtained during a real-life listening condition. FMRI data was re-analyzed from a previously published dataset of 18 musicians and 18 nonmusicians^11,13,25,26^ who were scanned during continuous listening to pieces of real music related to three distinct musical genres. Musical features were extracted computationally from the music pieces and used to encode regional neural activations in each participant. Log-likelihood ratios between musician and nonmusician models describing neural activation patterns arising from musical feature processing were used as input to a classifier that was trained to infer the musicianship of the participants. Model training and testing was done using cross-validation to avoid overfitting and to obtain a realistic estimate of the model performance on novel participants. Utilizing temporal information in classification rather than directly using correlations between stimulus features and neuroimaging data enables one to dynamically take into account the temporally changing statistical uncertainty. This has been found effective in past approaches on fMRI decoding^27,28^. However, these past approaches were designed for classification of data into experimental conditions rather than classifying participants in terms of demographic attributes.

The hypotheses of the present study are as follows: First, based on past findings of significant differences between musicians and nonmusicians, we expected to obtain higher-than-chance classification accuracies. However, since we were conducting the analysis on healthy participants, we did not expect to reach as high accuracy levels than those obtained for neurological disorders having severe structural or functional effects on the brain, such as autism spectrum disease or Altzheimer’s disease. Second, the brain regions expected to yield the highest discriminative power for the binary classification as a musician or nonmusician were areas related to the motor and auditory sensory systems^11,29–33^. Third, hemispheric asymmetries were expected to be observed on the basis of specialization in music processing for specific musical attributes evidenced in previous work^13,34–36^. Fourth, the decoding accuracy was expected to be driven mainly by high-level musical features, as neural processing of these features has been suggested to have high inter-participant variability^10^ and are dependent upon rules learned in exposure to music^11^.

## Results

### Classification Accuracy

First the decoding accuracy was examined as a function of the number of included regions in the model. The decoder was cross-validated using the concatenated stimulus for training and testing.

**Figure 1:**
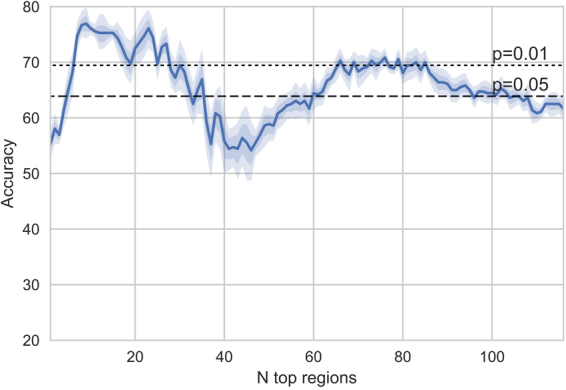
Accuracy (%; mean and 68% (dark color), 95% (light color) confidence intervals across the cross-validation runs) with different number of regions.

As can be seen in Fig. 1, a rather low number of top regions yields the optimal performance for the musical stimulus. The highest mean accuracy of 76.94% is achieved using nine regions (Sensitivity = 73.33%, Specificity = 80.56%, AUC = 0.8059). The statistical significance of the accuracies was examined by comparing the accuracies obtained from each cross-validation run to a binomial distribution (n = 36; p = 0.5) and taking the median of the p-values. Based on this, the obtained accuracy with nine regions is significantly (p < 0.0001) above the chance rate of 0.5 for this binary classification with equal number of participants in both classes. The *R*^2^ measures between predicted and actual BOLD time series for the linear regression models of each participant corresponded to correlations of 0.1 to 0.3.

As participants’ familiarity with the musical pieces differed between musicians and nonmusicians, additional analysis was done to rule out the effect of familiarity from the decoder model performance. To this aim, partial correlations were computed between the probabilities for the musicianship class obtained from the decoder and each participant’s actual class, removing the effect of each participant’s mean familiarity across the three musical stimuli. The mean correlation across the cross validation runs (r = 0.44) was significant at p < 0.01, and thus we conclude that the classification results do not reflect the participants’ familiarity with the musical pieces used in the experiment.

### Selected Regions and their Discriminative Power

The regions selected by the decoder models operating on nine regions, giving the optimal classification accuracy, were inspected. Since at each fold the selected nine regions are potentially different due to the use of different training data, the selected regions for the models from all of the 180 cross-validation folds were inspected. Nine regions were consistently selected: the right Superior temporal gyrus (STG); bilateral Caudate nucleus (CAU), the right Middle frontal gyrus (MFG), the orbital part of the right MFG, bilateral Anterior cingulate and paracingulate gyrus (ACG), and the triangular and opercular part of the right Inferior frontal gyrus (IFG). Right lateralization was observed in the selected regions–seven out of the nine regions were located at the right hemisphere.

**Figure 2:**
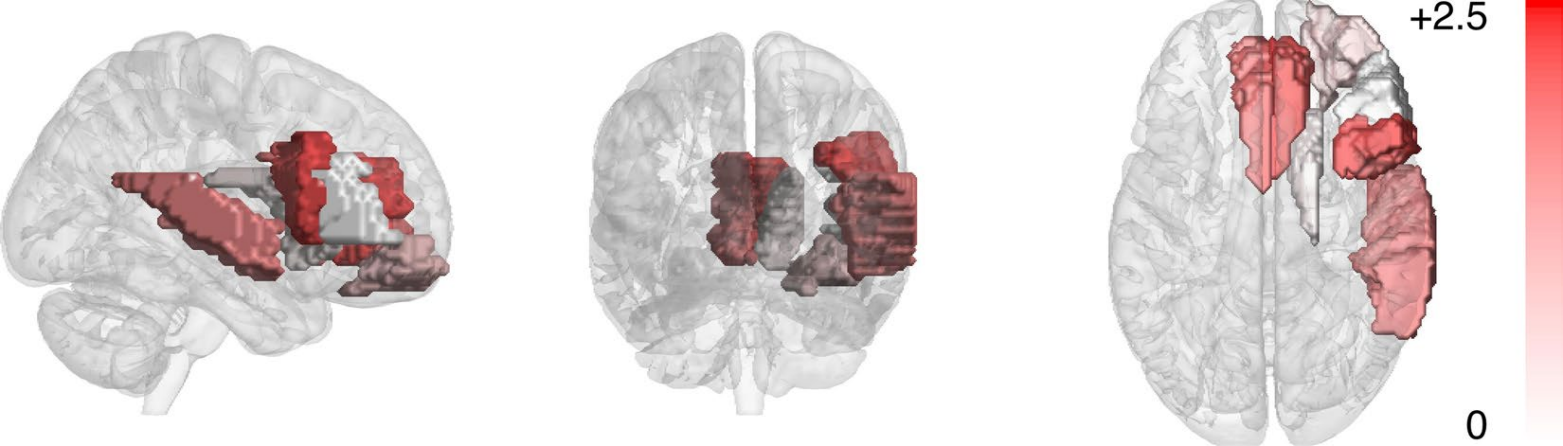
Musicianship discrimination in the nine discriminative regions–Z-values for the group differences of log-likelihood ratios averaged across cross-validation runs.

To estimate how well the nine selected regions individually discriminate musicians and nonmusicians, the log-likelihood ratios were computed for all participants using the cross-validation procedure. This is equivalent to computing the input features for the classification stage of the decoder. For each cross-validation run, the obtained values were compared between musicians and nonmusicians using the one-tailed Student’s T-test (df = 35; one-tailed test was used since log-likelihood ratios between musicians and nonmusicians can be assumed to be higher values for musicians than nonmusicians). The Z-values thus computed were averaged across the cross-validation runs, and the p-value was computed from the average Z-value. The statistics are shown in Table 1, and the Z-values are visualized in Fig. 2. Regions yielding statistical significant differences at p < 0.05 for the discrimination were the bilateral ACG, the opercular part of the right inferior frontal gyrus (IFG), and the right superior temporal gyrus (STG).

**Table 1:** Z-values and p-values for the log-likelihood ratios averaged across the cross-validation runs (p < 0.05 in boldface).

Region	Z-value	p-value
Caudate nucleus L	−0.122	0.549
Caudate nucleus R	0.428	0.334
Middle frontal gyrus R	0.008	0.497
Middle frontal gyrus, orbital part R	1.000	0.159
Anterior cingulate and paracingul. gyrus L	1.901	**0.029**
Anterior cingulate and paracingul. gyrus R	2.229	**0.013**
Inferior frontal gyrus, opercular part R	2.225	**0.013**
Inferior frontal gyrus, triangular part R	0.299	0.382
Superior temporal gyrus R	1.654	**0.049**

Although the selected regions were right-lateralized, the ranking-based region selection could have discarded the homologous regions in the left hemisphere by only slight margins. Therefore, the lateralization of the discriminative power of the significant regions was tested by means of comparison of the decoding accuracies obtained with the regions at the right and left hemispheres. To this aim, the decoding accuracy was computed by training the decoder model separately using homologous regions of ACG, opercular part of the IFG (IFGoper), and STG from the left and right hemisphere as well as bilaterally. Again, the cross-validation was run ten times, and the obtained accuracies were averaged across the runs. The results are displayed in Table 2. For both the ACG and IFGoper, the right hemispheric regions yielded the highest accuracy 66.94% and 64.17%, respectively. These accuracies were significantly higher than the chance rate at p < 0.05. The STG did not yield accuracy higher than the chance rate with any of the region configurations.

**Table 2:** The average decoding accuracy (%) using the regions from the left (LH), right (RH), and both hemispheres. The statistical significant differences to the chance classification rate are typed in boldface.

Region	LH	LH + RH	RH
ACG	57.78	61.39	**66.94**
IFGoper	48.06	56.67	**64.17**
STG	50.83	57.78	56.94

### Musical Feature Contributions

**Figure 3:**
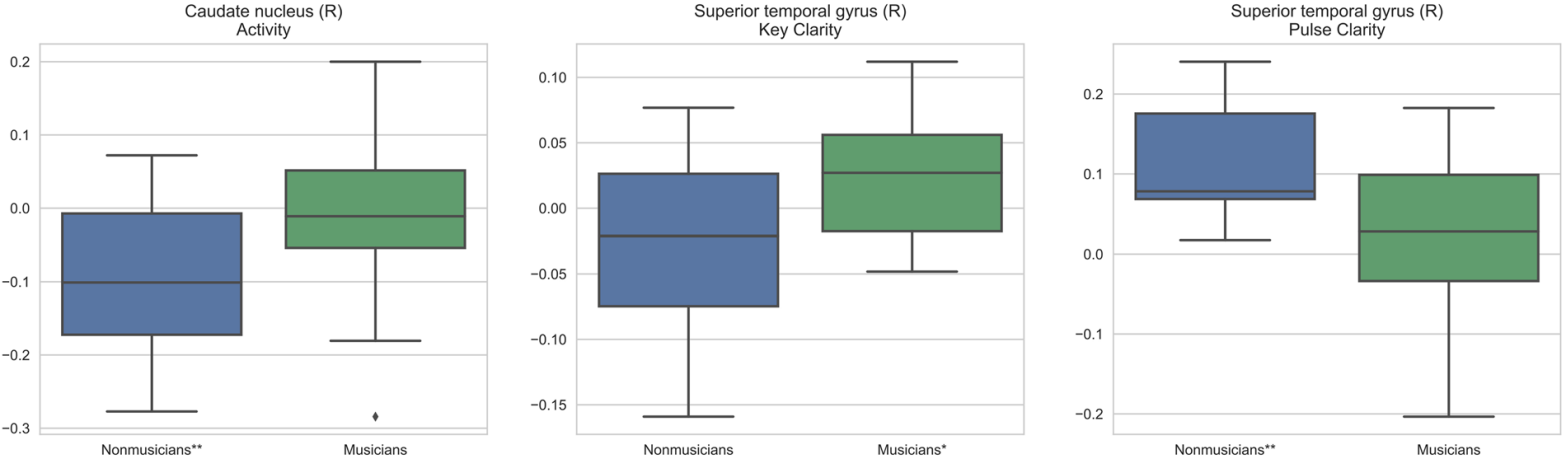
Distributions of feature beta coefficients for region/feature combinations yielding the most significant differences between the group means as shown by the two sample t-tests. Significance from one sample t-tests for the groups are marked with ^*^(p < 0.05) and ^**^(p < 0.01) after the group labels.

The standardized musical feature beta coefficients from the participant-specific linear regression stage of the modeling were inspected to see whether the musical features activate the nine discriminative regions differently for musicians than nonmusicians. Two sample t-test statistics, assuming independent samples, were computed for the differences of the mean coefficient values between musicians and nonmusicians. Figure 3 shows the group distributions of the coefficients for the most significant region/feature combinations based on these tests. It is notable that all of the obtained regions are located at the right hemisphere. For the right STG, Key Clarity coefficient values were higher for musicians (t = 2.49, p < 0.05), whereas Pulse Clarity values were higher for nonmusicians (t = −2.83, p < 0.01). For the right CAU, Activity values were higher for musicians (t = 2.35, p < 0.05). In order to back the claim for the group-specific effect of features on the region activations, one-sample t-tests were conducted for musicians and nonmusicians separately to see if the group means differ significantly from zero. The significance levels from these tests are shown in Fig. 3. The right CAU correlates negatively with Activity in nonmusicians, the right STG correlates positively with key clarity in musicians, and the right STG correlates positively with Pulse Clarity in nonmusicians.

## Discussion

The brain regions selected by the decoder which individually best discriminated between musicians and nonmusicians were the bilateral anterior cingulate and paracingulate gyrus (ACG), the opercular part of the right inferior frontal gyrus (rIFGoper, corresponding to the right homologue of the Broca’s area), and the right superior temporal gyrus (rSTG). Thus, these set of areas can be regarded as core areas in the processing of musical features which are most affected by musical training and consequently exhibit highest discriminative power amongst all brain areas.

The ACG is located in the medial surface of the frontal gyrus and constitutes a prominent node within the salience network, an intrinsically connected large-scale network which is implicated in a variety of complex brain functions by means of integrating sensory, emotional, and cognitive information^37,38^. The ACG is amongst the most frequently activated regions in all of functional neuroimaging research^39^, which is indicative of its central engagement in an array of different cognitive functions. It plays a crucial role in attentional control^40^, which is consistent with the observation of ACG’s increased activation when participants selectively attend or orient attention to an unexpected stimulus^41^.

In the context of music, the ACG seems to exhibit larger BOLD responses bilaterally in musicians compared to controls as reflective of enhanced music-related working memory capacity, suggesting musicians’ ability to maintain focus on task-relevant stimuli, likely as a result of their training as music experts^42^. The ACG has also been found to covary positively with the degree of prediction during sensorimotor synchronization in musicians^43^. The present music-listening paradigm involves a sustained attention task, in which musicians, by virtue of their expertise, may have been more engaged than nonmusicians as a result of their increased sensitivity to the musical material. This could explain the recruitment of this region as one of the discriminative regions selected by the decoder.

The opercular part of the IFG (BA44) as a central structure in music processing is well-supported by the converging evidence from the literature dealing with musical discrimination, harmony, timbre, tonality, rhythm, intensity, and error detection for melody and harmony during score reading^44^. Specifically, the rIFGoper is the right hemispheric homologue of Broca’s area, a well-recognized neural substrate for speech production and grammar acquisition^45^.

Previous work has revealed that the frontal operculum, with a right hemisphere asymmetry, constitutes a sensitive area involved at least partly in the processing of musical syntax using chord sequence and melody paradigms^46,47^. Similarly, activation of the rIFGoper has been observed during music listening by an increasing degree of auditory working memory operations^48,49^. Moreover, differential activity in the rIFGoper region was elicited when participants were asked to spontaneously generate melodic phrases vs linguistic sentences^50^. In general, activations within the rIFGoper seem to underpin processes of music–syntactic analysis and detection of violations in the harmonic rules of music, which in turn requires working memory resources^51^, suggesting that musical syntax may be processed in a homologous manner to language syntax.

In terms of effects of musical training, group comparisons have determined that bilateral IFGoper together with the anterior rSTG become more strongly activated in musically trained than untrained individuals in response to music-syntactically irregular chords^46^. This has been taken to imply a more prominent involvement of neural resources engaged in music-syntactic processing in musicians, as a consequence of the enhanced representations of musical regularities bolstered by years of experience in both music practice and theory. This acquired musical fitness would purportedly render them more sensitive to react to the violations of these regularities^46^. Moreover, correlation analysis has revealed that the earlier the onset of musical training, the higher the node degrees (higher connectivity with other areas) in the right Broca’s homologue^26^. This suggests that plasticity in this area is associated to intense musical training, particularly at an early onset age. On the basis of the literature cited above, the ability of the decoder to extract this particular syntax-processing core region in search for the optimal classification accuracy may support right Broca’s homologue’s sensitivity to the impact of musical training on the processing and detection of regularities in music.

The bilateral STG is the site of the primary and association auditory cortex, responsible for sound perception. Its right hemispheric homologue (rSTG) exhibits an improved ability to resolve spectral information compared to its left hemisphere counterpart^52,53^. Thus, it is considered to be dominant for representing melody, pitch, harmony, and timbre^54^. Robust supporting evidence on how the two hemispheres integrate distinct aspects of auditory information to make sense of the auditory input derives from both lesion studies with unilaterally brain-damaged patients^55,56^, reporting that right temporal lesions cause amusia or deficits in the discrimination of melodies), and neuroimaging studies^53^. Moreover, rSTG has been implicated as a core region for processing musical features from naturalistic music stimuli, with activation patterns that generalize across musical pieces^9^.

Formal musical training endows musicians with explicit and implicit knowledge about musical categories. This impacts the musician’s discrimination abilities compared to the untrained listener in terms of the detection and analysis of acoustic events, such as intervals, harmonic types and chord progressions^57^. As a result, the rSTG would manifest a high discriminative accuracy with the present decoding approach to separate musicians’ brain responses from those of nonmusicians in continuous music listening, drawing from long-term experience-driven plasticity in the musical domain.

The direction of the lateralization observed in the present decoding approach involving right fronto-temporal areas is consistent with the right hemispheric dominance of frontal and temporal regions for the processing of specific acoustic characteristics of music (and also certain pragmatic aspects of language, i.e., prosodic or intonational information expressed by accentuation and boundary markings through pitch variation^58^). In music, the right-left dissociation responds to different attributes of acoustic information. For example, temporal modulations increase activation in the auditory cortical region in the left hemisphere, whereas spectral modulations do so in the homologous right auditory cortical region^53,59^. Similarly, the decoder’s selection of the right-hemispheric frontal operculum for accurate decoding of musicianship conforms with its central role in the syntactic processing of music and thus sensitive to training-driven plasticity. Consistent with this, right-lateralized responses to music have been observed in previous findings on musical processing in musicians during listening^1,9,10,47^. Similarly, the decoder’s selection of the right-hemispheric frontal operculum for accurate decoding of musicianship conforms with its central role in the syntactic processing of music, thus sensitive to training-driven plasticity.

So far we have discussed the contribution of all musical features taken together. To better describe the information that is used by the classifier to separate musicians and nonmusicians, we investigated the contributions of each of the six individual musical features for the selected regions. The three musical dimensions and regions that showed the most significant differences in their beta coefficients in the encoding stage of the present approach were Pulse Clarity (rSTG), Key Clarity (rSTG), and Activity (right caudate nucleus [rCAU]). We discuss these features in the following.

Pulse Clarity yielded a significant discriminative power and had significantly higher than zero beta coefficients for nonmusicians for the rSTG. The STG has been established in the literature as a neural substrate for rhythm in controlled experimental setting^60^, but also under naturalistic stimulation, where the activation exhibited a rightward bias^1,9^. Results are in line with past findings with the same fMRI and musical feature data as used here, where the rSTG was observed as part of a functional network subserving pulse clarity in nonmusicians to a greater degree than in musicians^11^. They hypothesized that models of pulse clarity based on the acoustic properties of the stimulus may predict better nonmusicians’ brain responses to the musical pulse than those of musicians. A reason for this may be that musicians’ models of pulse clarity depend less on acoustic features and more on rules of metricality, learned through musical training, than those of nonmusicians^11^. Thus, this could explain the efficient discrimination of musicianship class by this musical feature.

Similar to Pulse Clarity, Key Clarity is a complex percept dependent on top-down, cognitive control processes and is therefore expected to be more individualized and thus display higher inter-subject variance compared to low-level acoustic features^61^. This has also been suggested by high inter-subject variance in the decoding accuracy^10^. Key Clarity yielded a significant discriminative power in the rSTG and higher than zero beta coefficients for the rSTG region for the musically trained listeners. This indicates that brain responses related to the percept of tonality may be either more homogeneous or alternatively stronger across musicians than nonmusicians. In addition, the fact that Key Clarity shows significant differences in rSTG is in line with previous findings relating Key Clarity with the primary right auditory cortex as the specialized brain substrate in processing tonality, in particular consonant chords^62^.

Activity, as a low-level percept, may be more stable and less individualized across participants compared to Pulse and Key Clarity. Perceptually, music high in Activity is characterized by high degree of dissonance and rapid high frequency changes (e.g., quick passages in the music featuring multiple instruments playing different pitches simultaneously). Brain responses in the rCAU, part of the dorsal striatum within the basal ganglia structures, had significantly positive coefficients related to Activity in musicians. The CAU is involved in a wide range of functions related to motor control, cognition (memory, learning) and emotion. With reference to music, previous work suggests that distinct parts of the basal ganglia may touch upon specific aspects of music processing, where the dorsal region has a role in rhythm^60,63,64^. Other work relates the rCAU with working memory functions for music^49^ or with processing of sad and implicit musical emotions^65^. The rCAU has been found to be modulated by the emotional content of music, in particular, by high arousal emotions irrespectively of their valence (tension, joy and power)^66^. Closely related to these findings are results showing that the activation in the rCAU in anticipation to peak emotional responses to music correlated with dopamine release within the rCAU^67^. In view of the many functions of the CAU, it is difficult to assess the significance of this result. However, in the light of the above cited literature suggesting a role of the rCAU in arousal and strong emotional responses in music, we hypothesize that Activity may represent aspects of the arousal dimension used in emotion research thus providing an explanatory context for this finding. According to this, musical training would seem to play a role in enhancing affective responsiveness to Activity.

We note that while the inferences made a posteriori based on the recruited regions found in this observational decoding study are speculative (being drawn from findings derived from previous literature), they serve to stimulate future ad-hoc hypothesis-driven studies. The musical features at temporal resolution of one second, as used here, have been found to be related to the BOLD response in past studies^1,9–11^. For allowing to study the correlation between features and BOLD signal, the low-level features were down-sampled to one second, but the typical frame for calculating them is around 30 msec. While we are aware that the temporal dynamics of acoustic features, especially the low-level timbral ones, is much faster than the BOLD signal, our previous findings demonstrate that the hemodynamic response can track the temporal course of the features at 1-sec scale.

In sum, despite the existence of inter-subject variability independent from musical training itself (e.g., personal preference or musical background), it was possible to find the universal within-group representation of music processing with enough accuracy for a successful classification. Furthermore, it could be assumed that bottom-up mechanisms (low-level acoustic features) would be mainly driving within-group representations of music. Conversely to this, we observed that two top-down features, namely Key Clarity and Pulse Clarity, were driving within-group representations in musicians and nonmusicians, respectively.

## Conclusions

We demonstrated the feasibility to decode musicianship class from how individual brains listen to real music, attaining a classification performance comparable to current state-of-the-art results in other classification tasks, including patients’ classifications. Additionally, we found that, amongst all musical dimensions used, high-level (tonal and rhythmical) features (i.e., Key and Pulse Clarity) yielded the highest discriminative power followed by low-level (timbral) features. The processing of these high-level percepts would seem to be most influenced by listeners’ previous musical exposure and experience. Furthermore, the discriminative brain substrate in relation to tonality and rhythm was the rSTG, consistent with the auditory right-hemispheric asymmetry underlying music processing found in previous work.

The present work provides a framework for identifying core regions in music processing affected by musical training. It reveals the existence of musicianship-specific representations of music processing during realistic listening, demonstrating the potential of naturalistic settings in combination with fMRI measures for brain-reading paradigms. At the same time, this approach advances the study of demographic decoding in the field of brain imaging in general. Future directions will benefit from the inclusion of additional musical stimuli and a larger participant pool, which can include different types of musicians. This could potentially reveal aspects of the specificity of instrument-driven plasticity.

In this approach, it was possible for the decoder to categorize participants into classes by efficiently extracting the information present in coarse activation patterns, averaged over large anatomical areas, that do not necessary reflect functional demarcations. Such coarse parcellation of the brain represents one limitation of the present approach, whereby voxel activity was averaged across 116 anatomical regions (according to the AAL atlas^68^). This was required in initial stages of the approach to reduce the dimensionality of the fMRI data beneficial to trading off between overfitting and generalization, while at the same time reducing the computational load. In the future, more subtle distinctions between classes could be inferred by using a more refined parcellation of the anatomical brain volume. Such a finer-grained approach could potentially reveal subtle functional differences observed within the regions herein investigated. Another possibility could be to use group-wise parcellations derived in a data-driven manner by means of e.g., Independent Component Analysis (ICA)^69^. According to this, different parcels could be obtained that delineate clear changes in the functional connectivity profile. This has the additional advantage of providing rich information regarding the hierarchical structure of the functional organization of the brain, which may lead to an increased decoding accuracy. One option would also be to focus on particular areas of the brain such as frontal and temporal lobes and select regions from those areas prior to the modeling process.

The novelty of this decoding approach is the integration of MIR, namely computational acoustic feature extraction, with functional neuroimaging measures obtained in a realistic music-listening environment. Thus, it represents a significant contribution that complements recent brain-reading methods to decode stimulus information from brain activity in realistic conditions in the auditory and visual domains^70,71^. Based on their weight distributions, the most critical brain regions for discriminating between musicianship class were the rSTG (locus of the auditory cortex), rIFCoper (locus of the right Broca’s area), and bilateral ACG (a key node in the salience network). Because these areas were crucial in decoding class-specific hemodynamic responses, they can be regarded as core structures in music processing which are most affected by intensive, lifelong musical training.

## Methods

### Participants

The participant pool consisted of 18 musicians and 18 nonmusicians, comprising groups comparable with respect to gender, age distribution, cognitive measures (Processing Speed and Working Memory Index Scores from the WAIS-WMS III^72^), and socioeconomic status (Hollingshead’s Four-Factor Index^73^). The pool is the same as in several previous studies^11,13,25,26^. The participants were labeled as musicians or nonmusicians based on self-reports and information obtained with questionnaires, included in Helsinki Inventory of Music and Affective Behaviors (HIMAB)^74^. Those labeled as nonmusicians were required to have less than 5 years of music training, hold no official music degree, to not identify themselves as musicians, and to not have earned money for playing. The musician group was homogeneous in terms of the duration of their musical training, onset age of instrument practice, and amount of years of active instrument playing. Musicians’ main instruments were strings (n = 7), piano (n = 8), wind (n = 2), and mixed (n = 1). For details, see Table 3).

**Table 3:** Participant demographics (Mus = musicians, NMus = nonmusicians).

group	N	age	gender	hand	soc-eco status	WAIS-III PSI	music listening (h/week)	musical training (years)	instrument playing (years)
Mus	18	28.2±7.8	9F	18R	43.6	116.3	18.2±11.2	15±4.7	21.2±6.2
NMus	18	29.2±10.7	10F	17R	35.4	115.7	12.4±6.7	1.6±2.2 (n = 8)	2.1±3.0 (n = 9)

### Musical Stimuli

Three non-vocal musical pieces were used as stimuli in the experiment:

DreamTheater Petrucci, J., Myung, J., Rudess, J. & Portnoy, M. (2003). Stream of Consciousness (instrumental). [Recorded by Dream Theater]. On *Train of Thought [CD]*. Elektra Records. (2003); Spotify link: http://open.spotify.com/track/3TG1GHK82boR3aUDEpZA5f; Excerpt: 0–07:50.979.

Piazzolla Piazzolla, A. (1959). AdiÓs Nonino. [Recorded by Astor Piazzolla y su Sexteto]. On *The Lausanne Concert [CD]*. BMG Music. (1993); Spotify link: http://open.spotify.com/track/6x5SzbloyesrQQb3Ht4Ojx; Excerpt: 0–08:07.968.

Stravinsky Stravinsky, I. (1947). The Rite of Spring (revised version for Orchestra) Part I: The Adoration of The Earth (Introduction, The Augurs of Spring: Dances of the Young Girls, Ritual of Abduction). [Recorded by Orchestra of the Kirov Opera, St. Petersburg - Valery Gergiev]. On *Stravinsky: The Rite of Spring/Scriabin: The Poem of Ecstasy [CD]*. Philips. (2001); Spotify link: http://open.spotify.com/album/22LYJ9orjaJOPi8xl4ZQSq (first three tracks); Excerpts: 00:05–03:23, 0–03:12, 0–01:16 - total duration: 07:47.243.

These stimuli, each about 8 min in duration, cover three distinct genres of progressive rock, Argentinian tango, and 20th century classical music, respectively.

### Experimental Procedure

Participants’ brain responses were acquired using fMRI while they listened to three musical stimuli in a counterbalanced order. For each participant, the stimuli loudness was adjusted to a comfortable but audible level inside the scanner room (around 75 dB). In the scanner, participants’ task was to attentively listen to the music delivered via high-quality MR-compatible insert earphones while keeping their eyes open. Moreover, participants were instructed that after listening they would be asked some questions about the musical pieces heard. This task was aimed at obtaining affective ratings on the music and keeping the attention focused on listening (for an analysis of fMRI data combined with affective ratings, see Alluri *et al*.^25^). The participants also reported their familiarity with the musical stimuli used in the experiment on a scale from one (not familiar) to five (very familiar). After averaging each participant’s familiarity rating across the stimuli, musicians showed higher familiarity with the stimuli (Mean = 2.96, STD = 1.21) than nonmusicians (Mean = 2.07, STD = 1.03). We took this factor into account in the decoder evaluation. The fMRI data employed in the current study has been used previously^11,13,25,26^.

All experimental procedures, part of a broader project called “Tunteet” initiated and coordinated by E.B., were approved by the Coordinating Ethics Committee of the Hospital District of Helsinki and Uusimaa. All procedures were conducted in agreement with the ethical principles of the Declaration of Helsinki. All participants signed an informed consent on arrival to the laboratory and received compensation for their time.

### fMRI Scanning and Preprocessing

A 3T MAGNETOM Skyra whole-body scanner (Siemens Healthcare, Erlangen, Germany) and a standard 20-channel head-neck coil was used to acquire single-shot gradient echo planar images (EPI) every two seconds (33 oblique slices, field of view = 192 × 192 mm; 64 × 64 matrix; slice thickness = 4 mm, interslice skip = 0 mm; echo time = 32 ms; flip angle = 75°), providing a whole-brain coverage. T1-weighted structural images (176 slices; field of view = 256 × 256 mm; matrix = 256 × 256; slice thickness = 1 mm; interslice skip = 0 mm; pulse sequence = MPRAGE) were collected for individual coregistration.

Functional MRI scans were preprocessed using SPM8 (Statistical Parametric Mapping) and VBM5 for SPM. For each participant, the images were realigned, spatially normalized into the Montreal Neurological Institute template (12 parameter affine model, segmentation: gray matter, white matter, and cerebrospinal fluid; realignment: translation components <2 mm, rotation components <2°), and spatially smoothed (Gaussian filter with FWHM of 8 mm). Movement-related variance (based on three translation and three rotation components) in the fMRI time series were regressed out from each voxel time series to minimize motion artefacts. Following this, the data was detrended using spline interpolation and temporally smoothed using a Gaussian kernel with a width of 4 s.

In the present study, in order to reduce the computational load, decoding model complexity, and overfitting issues in decoding model training, dimensionality of the fMRI data was reduced. The data was parcellated into 116 whole brain anatomical regions based on the Automated Anatomical Labeling (AAL) atlas^68^ by averaging the time-series over all voxels within each region.

### Musical Feature Extraction

We used the approach implemented by Alluri *et al*. for musical feature extraction^1^. Frame-based features related to timbre (20 features; frame length = 25 ms; overlap = 50%), rhythm (3 features; frame length = 3 s; overlap = 67%), and tonality (2 features; frame length = 3 s; overlap = 67%) were extracted from the stimuli using the MIR Toolbox^3^. The resulting feature time-series were made compatible with the fMRI data by a series of processing stages. The features were convolved with a double-gamma hemodynamic response function (peak = 5 s; undershoot = 15 s) and detrended similarly to the fMRI data. The obtained features were then downsampled to match the sampling rate of the fMRI scans. Finally, the features were mapped to six varimax-rotated principal components that were defined and perceptually validated by Alluri *et al*.^1^.

The following lists the musical features and the respective maximum feature loadings. **Fullness**: spectral fluctuations at low frequencies (50–200 Hz); **Brightness**: spectral rolloff (right-skewedness of the power spectrum) and spectral centroid; **Activity**: the degree of roughness (i.e., sensory dissonance) and spectral fluctuations at the high frequencies (above 1600 Hz); **Timbral Complexity**: flattness of the sound spectrum; **Pulse Clarity**: the strength of rhythmic periodicities sound, representing how easily the underlying pulsation in music can be perceived. **Key Clarity**: the strength of the estimated key, computed as the maximum of cross-correlations between the chromagram extracted from the music and tonality profiles representing all the possible key candidates.

The timbral features (Fullness, Brightness, Activity, and Timbral Complexity) represent low-level percepts as they depend on early perceptual processing mechanisms, whereas Pulse and Key Clarity represent high-level percepts, requiring knowledge based on our previous exposure to music.

### Decoding Approach

**Figure 4:**
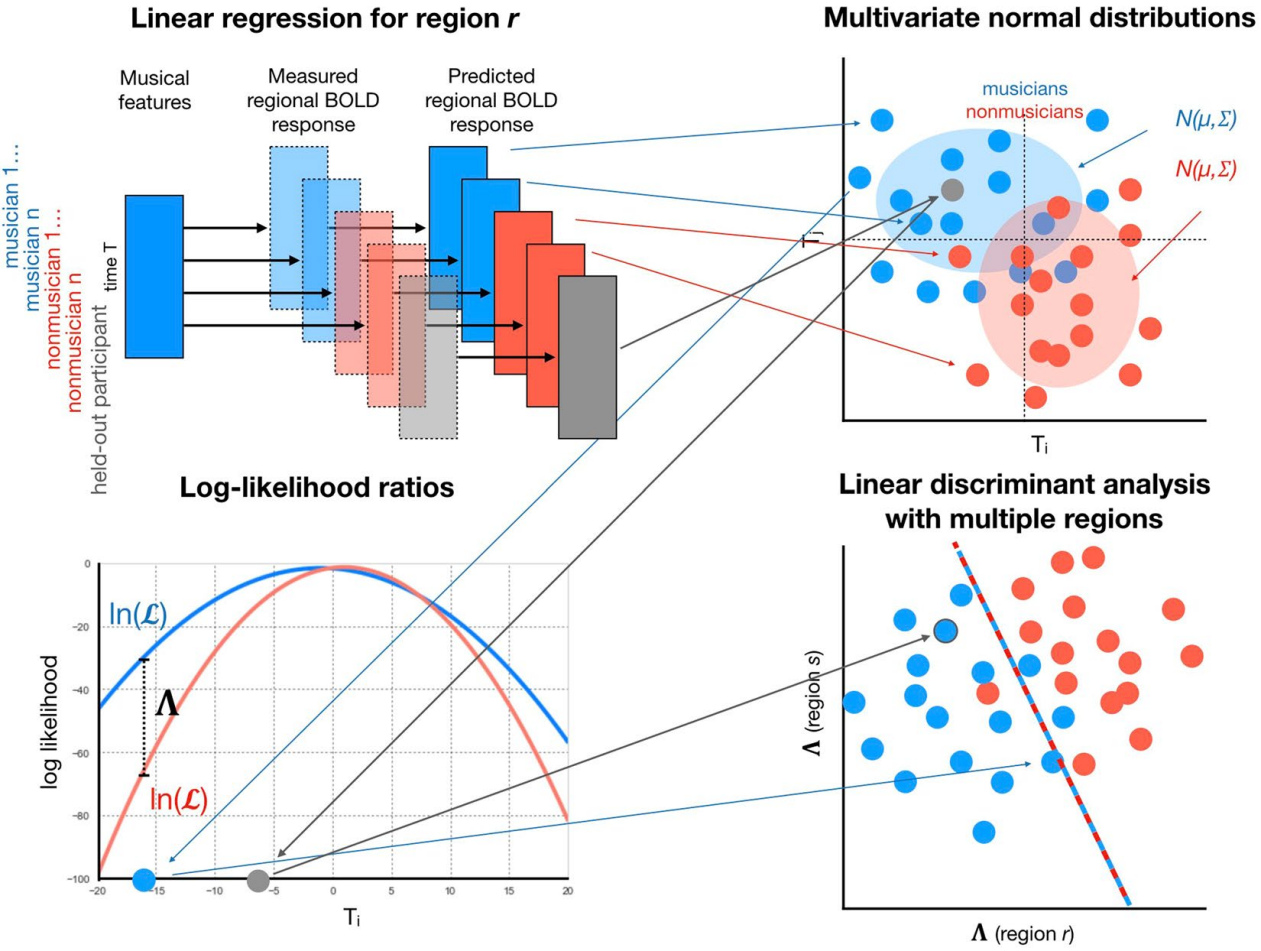
Different stages of the decoder training: participant-specific region time series encoding with linear regression, statistical musicianship group modeling with multivariate normal distributions, feature extraction with log-likelihood ratios between groups; and classification. The first three stages are employed for multiple regions, and classicifation is done based on the obtained features related to these regions. The region selection stage is excluded in the visualization. An example participant held out from the decoder training, shown in grey, is classified as a musician.

The purpose of the decoding approach is to automatically detect discriminative neural processing patterns of musical features between musician and nonmusician classes, enabling one to use those patterns to classify a participant not used in the model training into either class with a specific statistical certainty. The decoder training involves multiple stages, outlined in Fig. 4: participant-specific region time series encoding with linear regression, statistical musicianship group modeling with multivariate normal distributions, feature extraction with log-likelihood ratios between groups, and final classification. Moreover, region selection was employed to reduce the dimensionality of the data while retaining those regions most effective for discrimination. In the following, we formally describe the stages of the automated decoding process.

Separately for each region, we consider two hypotheses *H*_*M*_: a participant belongs to the musician group *M*; and *H*_*N*_: participant belongs to the nonmusician group *N*. The contributions of the musical features to each participant’s region activations are estimated by linear regression

1 $${\bf{y}}\approx {\bf{X}}\beta ,$$

where **X** is a matrix composed of z-score transformed length *k* musical feature time series, **y** is a z-score transformed BOLD time series of a region, and *β* is the matrix of the standardized coefficients, estimated for each participant separately. The resulting approximation of **y** can then be represented in a *k*-dimensional vector space as

2 $$\hat{{\bf{y}}}=({\hat{y}}_{1},\mathrm{...}\,{\hat{y}}_{k})\in {{\mathbb{R}}}^{k},$$

where *k* denotes the number of time points. These signals are then treated as joint Gaussian random vectors in that space. In the following, *G* ∈ {*M*, *N*} denotes either the musician or the nonmusician group, and the modeling stages are employed similarly for both groups. To test the hypotheses *H*_*G*_, the distributions of the vectors for the musician and nonmusician groups are modeled as multivariate normal distributions by including the participants in the respective groups:

3 $${\hat{{\bf{y}}}}^{G}\sim {\mathscr{N}}({\mu }^{G},{{\rm{\Sigma }}}^{G}),$$

where ${\mu }^{G}=({\mu }_{1}^{G},\mathrm{...},{\mu }_{k}^{G})$ is a vector consisting of the mean for each time point, and ${{\rm{\Sigma }}}^{G}={\sigma }_{ij}^{2}$ is the covariance matrix between time points *i* and *j* (*i*, *j* ∈ [1, …, *k*])). We assume that ${{\rm{\Sigma }}}^{G}$ is a diagonal matrix so that the components of the random vector are independent. This is potentially a simplistic assumption since serial correlation in the means and variances might be present. However, using a full covariance matrix, although it might improve the results, would require fitting too many model parameters compared to the size of the data.

Given a group model, the log-likelihood for each participant’s predicted region time series is obtained by

4 $$\mathrm{ln}({ {\mathcal L} }^{G})=-\frac{1}{2}(\mathrm{ln}(|{{\rm{\Sigma }}}^{G}|)+{(\hat{{\bf{y}}}-{\mu }^{G})}^{T}{({{\rm{\Sigma }}}^{G})}^{-1}(\hat{{\bf{y}}}-{\mu }^{G})+k\,\mathrm{ln}(2\pi )),$$

where *T* indicates the matrix transpose. To estimate how likely a participant’s data fits the musician group compared to nonmusician group, the log-likelihood ratio is computed:

5 $${\rm{\Lambda }}=\,\mathrm{ln}({ {\mathcal L} }^{M})-\,\mathrm{ln}({ {\mathcal L} }^{N}).$$

The process is repeated for each region and the log-likelihood ratios are used as features for multivariate classification to jointly predict a participant as a musician or a nonmusician.

In general, a high ratio between the number of predictors (regions) and the number of samples (participants) tends to decrease the generalization of a classifier due to overfitting. In the present study, the number of available regions exceeds the number of participants, which is why region selection was employed prior to classification. Ranking-based subset selection was employed, wherein the regions were ranked according to how well the participants’ data fit the correct groups. This is estimated by taking the sum of log likelihoods for the correct groups across the participants:

6 $$\sum _{p\in M}\mathrm{ln}({ {\mathcal L} }_{p}^{M})+\sum _{p\in N}\mathrm{ln}({ {\mathcal L} }_{p}^{N}),$$

where ln $({ {\mathcal L} }^{M})$ and ln $({ {\mathcal L} }^{N})$ are the log-likelihoods for the musician and nonmusician groups, respectively. A specified number of top ranked regions was included from the ranked list of regions. The number of top ranked regions is considered here as a hyperparameter and its effect on classification performance is investigated.

Linear Discriminant Analysis (LDA) with singular value decomposition as solver (threshold 1.0e-4 use for for rank estimation) was used for the final classification. LDA was chosen as the classifier due to its simplicity and efficiency at handling linearly separable data. Moreover, in initial tests, nonlinear and linear Support Vector Machine models gave worse classification performance.

Linear Discriminant Analysis (LDA) was used to classify the data. LDA was chosen due to its efficiency at handling linearly separable data, and since the LDA model is effectively a simple matrix operation that allows straightforward interpretation.

LDA gives the estimated class for each sample (participant), but the probability that a sample belongs to a particular class can also be computed using the sigmoid function. In binary classification, the probability for the positive class, corresponding to the musician group in the present paper, can be computed by

7 $$P=\frac{1}{1+{e}^{-d}},$$

where *d* is the signed distance of a sample to the discriminant hyperplane, i.e., the distance is positive, negative, or zero if the sample is on the side of the positive or the negative class, or on the hyperplane, respectively.

To estimate the decoder performance on participants not used in decoder training, Eqs 1, 4, and 5 were applied on the participants’ data and the trained classifier was employed on the obtained likelihood ratios.

### Decoding Experiment

For the decoding experiment, the six musical features and each participant’s brain responses for the three musical stimuli were concatenated to obtain a general non-genre-specific stimulus with a total of ~24 minutes of data.

To estimate the accuracy of the decoder model, performance evaluation was conducted using 18-fold stratified cross-validation, where at each fold, one musician and one nonmusician were held out from the decoder training stages. The cross-validation was run ten times, each time on a different random split of the participants’ data. The decoder was trained and tested with different numbers of top regions, ranging from one to 116 (the number of regions in the AAL atlas).

### Data availability

The preprocessed, region-averaged BOLD data, preprocessed musical feature data, and participant musicianship and familiary information analyzed during the current study are available as supplementary material.

## Electronic supplementary material

Supplementary Dataset 1

Supplementary Dataset 3

Supplementary Dataset 2

Supplementary Dataset 4
