# Supplementary material for: Decoding Musical Training from Dynamic Processing of Musical Features in the Brain
Source: Sci Rep. 2018 Jan 15;8:708. doi: 10.1038/s41598-018-19177-5 (PMC5768727; doi:10.1038/s41598-018-19177-5)
Supplement: Supplementary file 1 — Supplementary Dataset 1 [file 41598_2018_19177_MOESM1_ESM.doc]

**Decoding Musical Training from Dynamic Processing of Musical Features in the Brain**

- date: 6/9/2017

- authors: P. Saari, I. Burunat , E. Brattico & P. Toiviainen

- affiliation: Department of Music, Art, and Culture Studies, University of Jyvaskyla, Jyvaskyla, PL 35(M), FI-40014 , Finland (Saari, Burunat, Toiviainen); Center for Music in the Brain (MIB), Department of Clinical Medicine, Aarhus University, Aarhus, DK-8000, Denmark (Brattico)

- contact: pasi.saari@jyu.fi

- citation: Saari, P., Burunat, I., Brattico, E. & Toiviainen, P. *Scientific Reports*. Decoding Musical Training from Dynamic Processing of Musical Features in the Brain.

**Description**

This dataset contains data that may be used to reproduce the results in the manuscript cited above.

**Data structures and formats**

All data is stored in comma-delimited csv files, with headers in the first row.

participants.csv (36 rows, 3 columns)

Participant musicianship class and reported familiarity averaged across the three musical stimuli.

- participant [integer]: participant id

- musician [integer]: 1=musician, 0=nonmusician

- familiarity [float]: familiarity value

BOLD.csv (37118 rows, 119 columns)

BOLD time series data after preprocessing and averaging into 116 regions in the AAL atlas (Tzourio-Mazoyer et al, 2002).

- stimulus [string]: musical stimulus, one of Dream Theater, Piazzolla, or Stravinsky

- participant [integer]: participant id

- time [integer]: time in seconds

- Amygdala (L),...,Vermis IX of cerebellum [float]: region-averaged BOLD activation value

musicalfeatures.csv (714 rows, 8 columns)

Musical feature time series data after preprocessing, convolution, and detrending.

- stimulus [string]: musical stimulus, one of Dream Theater, Piazzolla, or Stravinsky

- time [integer]: time in seconds

- activity,...,timbral complexity [float]: feature value

Musical Stimuli

Three non-vocal, approx. 8 minute musical pieces/excerpts were used as stimuli: Stream of Consciousness by Dream Theater, Adiós Nonino by Astor Piazzolla, and The Rite of Spring by Igor Stravinsky. For details, see the above citation.
